# The lysophosphatidylcholine-HIF-1α axis enhances apolipoprotein E sialylation and promotes electronegative LDL accumulation

**DOI:** 10.1016/j.isci.2026.116167

**Published:** 2026-06-05

**Authors:** Hua-Chen Chan, Hsiu-Chuan Chan, Liu-Fang Wang, Mei-Lin Chan, Wen-Chien Huang, Daniel Bender, Yu-Min Ko, Ming-Lung Yu, Guan-Ming Ke, Mei-Chuan Chou, Ching-Kuan Liu, Liang-Yin Ke

**Affiliations:** 1 Department of Medical Laboratory Science, College of Medical Science and Technology, I-Shou University, Kaohsiung 82445, Taiwan; 2 Department of Medical Laboratory Science and Biotechnology, College of Health Sciences, Kaohsiung Medical University, Kaohsiung 807378, Taiwan; 3 Department of Medical Research, MacKay Memorial Hospital, New Taipei City 104217, Taiwan; 4 Ph.D. Program in Life Sciences, College of Life Science, Kaohsiung Medical University, Kaohsiung 807378, Taiwan; 5 Department of Surgery, MacKay Memorial Hospital, Taipei 104217, Taiwan; 6 Graduate Institute of Biomedical Sciences, MacKay Medical University, New Taipei City 252005, Taiwan; 7 Institute of Pathology and Molecular Pathology, University Hospital Zürich, 8091 Zürich, Switzerland; 8 Hepatobiliary Division, Department of Internal Medicine, Kaohsiung Medical University Hospital, Kaohsiung Medical University, Kaohsiung 807378, Taiwan; 9 Graduate Institute of Animal Vaccine Technology, College of Veterinary Medicine, National Pingtung University of Science and Technology, Pingtung 912301, Taiwan; 10 Graduate Institute of Clinical Medicine, College of Medicine, Kaohsiung Medical University, Kaohsiung 807378, Taiwan; 11 Department of Neurology, Kaohsiung Medical University Hospital, Kaohsiung Medical University, Kaohsiung 807378, Taiwan; 12 Department of Neurology, Kaohsiung Chang Gung Memorial Hospital, Kaohsiung 807378, Taiwan; 13 Drug Development and Value Creation Research Center, Kaohsiung Medical University, Kaohsiung 807378, Taiwan; 14 Department of Laboratory Medicine, Kaohsiung Medical University Hospital, Kaohsiung 807378, Taiwan

**Keywords:** Cardiovascular medicine, Lipidomics

## Abstract

Electronegative low-density lipoprotein (L5-LDL) drives vascular injury in metabolic syndrome (MetS), yet its hepatic origin remains undefined. Here, we identify a lysophosphatidylcholine (LPC)-hypoxia-inducible factor-1α (HIF-1α) axis that governs apolipoprotein E (apoE) glycosylation and lipoprotein electronegativity. LPC activates HIF-1α in hepatocytes, inducing polypeptide *N*-acetylgalactosaminyltransferase (GALNT2) and ST3 β-galactodise α-2,3-sialyltransferase 1 (ST3GAL1) to promote apoE *O*-glycosylation and sialylation. This remodeling redirects lipoprotein uptake from the LDL receptor (LDLR) to lectin-like oxidized LDL receptor-1 (LOX-1), facilitating the formation of electronegative very low-density lipoprotein (V5-VLDL) and L5-LDL. Consistently, V5-VLDL from MetS subjects exhibits hyperglycosylated apoE mirroring L5-LDL. Disruption of this pathway attenuates apoE modification and LOX-1 engagement. These findings establish a mechanistic link between hepatic lipid remodeling and vascular inflammation and suggest a therapeutic strategy for targeting residual cardiovascular risk.

## Highlights

• Metabolic syndrome elevates electronegative V5-VLDL and L5-LDL
• V5-VLDL exhibits ApoE hyperglycosylation at T194 and T289
• LPC activates HIF-1α-dependent glycosylation in hepatocytes
• ApoE sialylation promotes LOX-1-mediated endothelial interaction

## Graphical abstract

**Figure:**
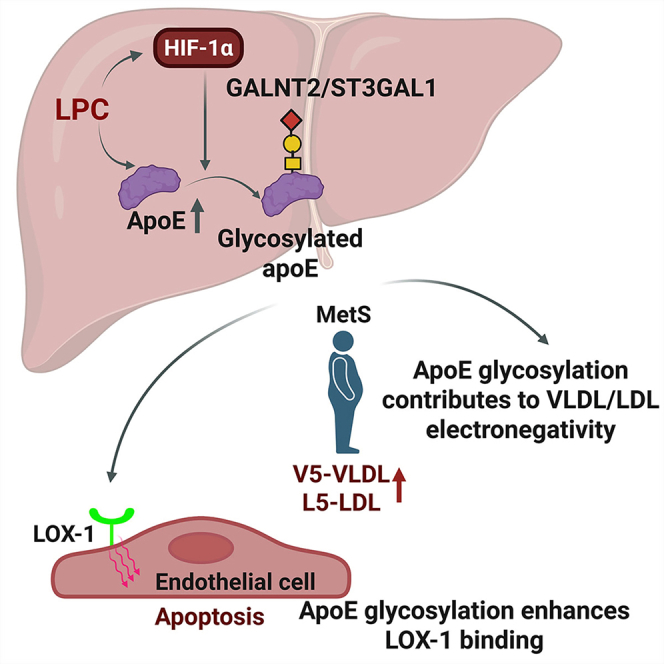

## Introduction

Apolipoproteins are synthesized predominantly in the liver and are essential for lipid transport, lipoprotein metabolism, and the regulation of plasma lipid homeostasis. Apolipoprotein B (apoB), the principal structural protein of very-low-density lipoprotein (VLDL) and low-density lipoprotein (LDL), transports cholesterol and triglycerides within lipoprotein particles.^1^ Apolipoprotein E (apoE), another key component of VLDL and LDL, facilitates the clearance of apoB-containing lipoproteins through interactions with the LDL receptor (LDLR).^2^^,^^3^^,^^4^^,^^5^^,^^6^

We previously reported that apoE with an isoelectric point (pI) of 5.5 is enriched in highly electronegative LDL (L5-LDL) compared with physiologic LDL (L1-LDL), implicating altered apoE charge in the formation of electronegative particles.^7^ In L5-LDL, apoE carries aberrant *O*-linked glycans—including *N*-acetylgalactosamine, galactose, and sialic acids—at serine 94 and threonine residues 194 and 289. These modifications increase the negative charge of apoE, reduce LDLR affinity, and enhance interaction with LDLR toward lectin-like oxidized LDL receptor-1 (LOX-1).^8^^,^^9^ Together with evidence linking excessive apoE sialylation to atherosclerosis,^10^ these findings suggest that dysregulated apoE glycosylation contributes to the formation of pathogenic lipoproteins, although the upstream drivers of this process remain poorly understood.

When LDL particles become highly electronegative, their receptor preference shifts from LOX-1, a scavenger receptor expressed on vascular endothelial cells. Activation of LOX-1 by electronegative LDL triggers endothelial dysfunction and apoptosis, in part through inhibition of the phosphatidylinositol 3-kinase/protein kinase B (PI3K/Akt) survival pathway.^9^ These observations highlight the importance of lipoprotein electronegativity as a pathogenic determinant of vascular injury.

VLDL particles are assembled in hepatocytes and secreted into the circulation, where they are progressively remodeled to LDL. Using anion-exchange chromatography, we previously fractionated human plasma VLDL and LDL into five subfractions (V1–V5 and L1–L5) and demonstrated that the most electronegative subfractions, V5-VLDL and L5-LDL, are markedly elevated in individuals with metabolic syndrome (MetS), cardiovascular disease, and myocardial infarction.^11^^,^^12^^,^^13^^,^^14^^,^^15^^,^^16^ These electronegative lipoproteins are highly atherogenic and induce endothelial injury through LOX-1-dependent signaling pathways. These findings led us to hypothesize that alterations in hepatic lipid metabolism promote the overproduction of electronegative V5-VLDL and L5-LDL.

Lysophosphatidylcholine (LPC) is a bioactive lipid mediator associated with hepatic inflammation, insulin resistance, and metabolic dysregulation in MetS.^17^^,^^18^^,^^19^^,^^20^ LPC can be generated from phosphatidylcholine by phospholipase A_2_ or produced during lipoprotein remodeling by enzymes such as lecithin-cholesterol acyltransferase and hepatic lipase.^21^^,^^22^^,^^23^ Metabolic disorders are frequently associated with hepatic LPC accumulation, increased LDL electronegativity, and progressive liver injury.^24^ Consistent with these observations, our lipidomic analyses revealed marked enrichment of LPC 16:0 in L5-LDL from individuals at high cardiovascular risk.^25^ Given the central role of the liver in VLDL biogenesis,^26^ these findings suggest that LPC-driven hepatic processes may influence the formation of electronegative lipoproteins.

We recently showed that LPC activates hypoxia-inducible factor-1α (HIF-1α) in trophoblast cells.^27^ HIF-1α is a key transcriptional regulator of cellular responses to metabolic stress and has been reported to regulate genes involved in lipid metabolism and glycosylation, including glycosyltransferases such as polypeptide *N*-acetylgalactosaminyltransferase (GALNT2) and ST3 β-galactodise α-2,3-sialyltransferase 1 (ST3GAL1).^28^^,^^29^^,^^30^^,^^31^^,^^32^ However, whether LPC-induced HIF-1α signaling contributes to the generation of electronegative lipoproteins through modulation of apoE glycosylation remains unknown.

In the present study, we propose that hepatic LPC accumulation activates an HIF-1α-GALNT2/ST3GAL1 pathway that promotes apoE *O*-glycosylation, thereby increasing lipoprotein electronegativity and enhancing interaction with endothelial LOX-1. By integrating human plasma analyses, mouse models, and mechanistic hepatocyte and endothelial cell experiments, this study identifies a previously unrecognized link between hepatic lipid remodeling and vascular dysfunction in MetS.

## Results

### ApoE glycosylation is elevated in V5-VLDL from patients with MetS

**Figure 1:**
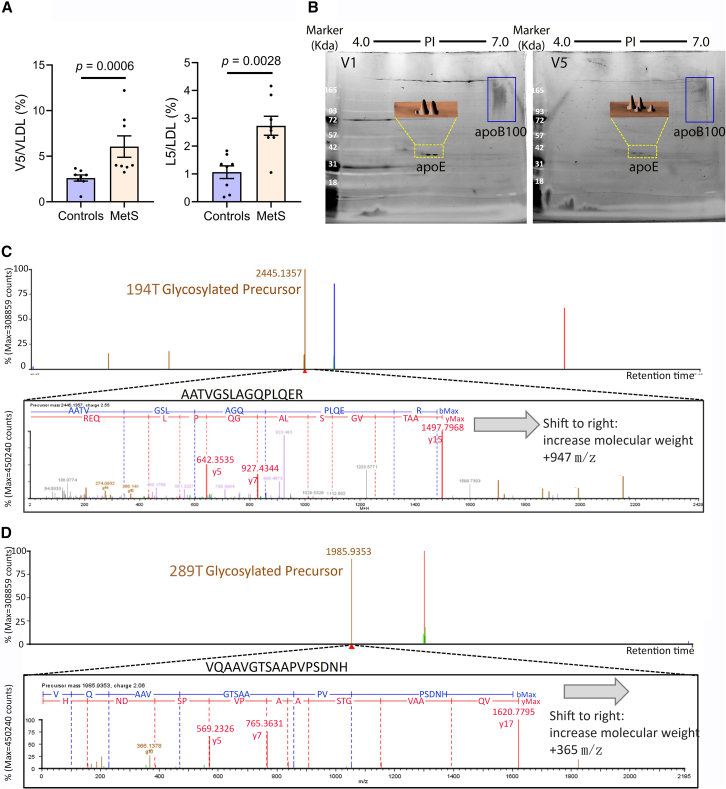
ApoE glycosylation is increased in V5-VLDL from individuals with MetS VLDL and LDL were isolated from fasting plasma of healthy controls and MetS subjects (*n* = 8 per group) by sequential ultracentrifugation. Lipoprotein subfractions were separated by anion-exchange fast protein liquid chromatography (FPLC) to quantify electronegative V5-VLDL and L5-LDL. ApoE glycosylation was analyzed by two-dimensional electrophoresis and LC-MS^E^. (A) Quantification of electronegative V5-VLDL and L5-LDL expressed as percentages of total VLDL or LDL. MetS subjects exhibited significantly higher proportions of both subfractions compared with controls (*n* = 8 per group). Data are presented as mean ± SD and analyzed using the Mann-Whitney U test. (B) Two-dimensional electrophoresis of equal amounts of V1 and V5 protein (2.5 μg). Proteins were separated by isoelectric focusing (pI 4.0–7.0, left to right) followed by sodium dodecyl sulfate polyacrylamide (SDS-PAGE). ApoE migrates at approximately 34 kDa. Compared with V1, V5 displays additional apoE isoforms shifted toward lower isoelectric point and slightly higher apparent molecular weight, consistent with increased glycosylation. Insets highlight the apoE region. (C) Liquid chromatography-data independent acquisition mass sepctronetry (LC-MS^E^) identification of a glycosylated apoE peptide (AATVGSLAGQPLQER) containing residue T194. The upper image shows the precursor ion detected by liquid chromatography-mass spectrometry (LC-MS), and the lower image shows the corresponding tandem mass spectrometry (MS/MS) fragmentation spectrum used for peptide sequencing. Annotated b- and y-ions confirm the peptide sequence. Sequential glycan-associated mass additions corresponding to *N*-acetylgalactosamine (+203 m/*z*), galactose (+162 m/*z*), and two sialic acids (+291 m/*z* each) indicate *O*-glycosylation at T194. (D) LC-MS^E^ identification of a second glycosylated apoE peptide (VQAAVGTSAAPVPSDNH) containing residue T289. As in (C), the precursor spectrum is shown above and the annotated MS/MS fragmentation spectrum below, confirming glycosylation at T289. Together, these analyses demonstrate enrichment of hyperglycosylated apoE species in V5-VLDL.

VLDL is synthesized in the liver and remodeled in the circulation to generate LDL. In patients with MetS, the proportions of electronegative V5-VLDL and L5-LDL were significantly higher than in healthy controls (V5-VLDL: 6.06% ± 1.18% vs. 2.61% ± 0.34%; L5-LDL: 2.7%3 ± 0.34% vs. 1.06% ± 0.23%; *p* = 0.0006 and *p* = 0.0028, respectively; Figure 1A). Because apoE in L5-LDL has previously been reported to contain *O*-linked glycans at residues T194 and T289,^9^ we examined whether V5-VLDL, the hepatic precursor of L5-LDL, exhibits similar apoE modifications. Two-dimensional electrophoresis revealed additional apoE isoforms in V5-VLDL compared with V1-VLDL (Figure 1B). These isoforms migrated toward a lower isoelectric point and slightly higher apparent molecular weight, consistent with post-translational modification such as glycosylation. To directly identify the modification sites, apoE peptides were analyzed by Liquid chromatography-data independent acquisition mass spectrometry (LC-MS^E^). As shown in Figures 1C and 1D, precursor ions corresponding to apoE peptides containing residues T194 and T289 were detected and subjected to tandem mass spectrometry (MS/MS) fragmentation. Annotation of the resulting b- and y-ion series confirmed the peptide sequences and localized the glycosylation sites. The spectra showed mass additions consistent with sequential attachment of *N*-acetylgalactosamine (+203 m/*z*), galactose (+162 m/*z*), and two sialic acids (+291 m/*z*), indicating *O*-linked glycosylation of these residues. Together, these findings suggest that hyperglycosylated apoE is enriched in V5-VLDL from MetS subjects, providing a structural basis for the increased electronegativity of these lipoprotein particles.

### Removal of sialylation attenuates lipoprotein electronegativity

**Figure 2:**
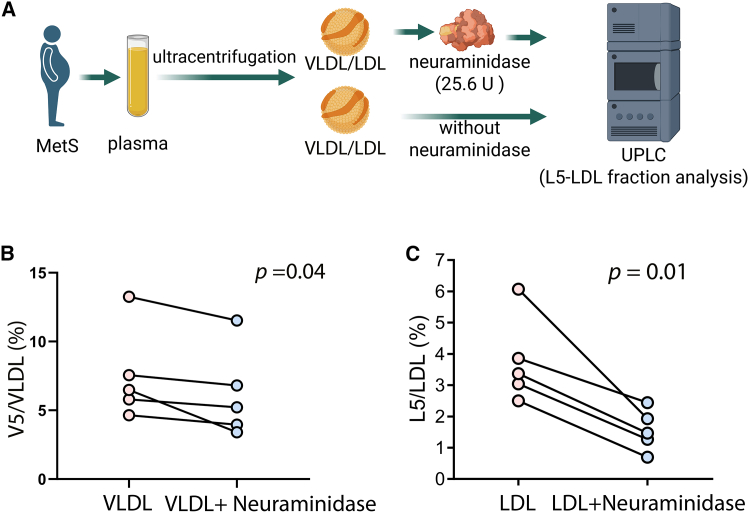
Desialylation by α2-3 neuraminidase reduces lipoprotein electronegativity (A) Experimental workflow. VLDL and LDL isolated from plasma of individuals with metabolic syndrome (MetS) were treated with α2-3 neuraminidase (25.6 U) or left untreated. Lipoprotein electronegativity was quantified by anion-exchange ultra-performance liquid chromatography (UPLC) fractionation to determine the relative abundance of electronegative V5-VLDL and L5-LDL fractions. (B) Paired comparison showing the reduction in V5-VLDL percentage after neuraminidase treatment (*n* = 5; *p* = 0.04). (C) Paired comparison demonstrating a decrease in L5-LDL percentage following neuraminidase treatment (*n* = 5; *p* = 0.01). Data are presented as mean ± SD. Statistical significance was determined using a paired *t* test.

Previous studies have associated increased apoE sialylation with enhanced lipoprotein electronegativity and altered receptor preference toward LOX-1.^8^ To examine whether enzymatic removal of terminal sialic acids modulates lipoprotein charge, VLDL and LDL isolated from patients with MetS were treated with 25.6 U α2-3 neuraminidase. Lipoprotein electronegativity was quantified by ultra-performance liquid chromatography (UPLC) using an anion-exchange column (Figure 2A).

Neuraminidase treatment significantly reduced the proportion of V5-VLDL from 7.54% ± 3.36% to 6.18% ± 3.26% (*p* = 0.04; Figure 2B). A similar effect was observed for LDL, where the L5-LDL fraction decreased from 3.77% ± 1.38% to 1.56% ± 0.66% following neuraminidase treatment (Figure 2C). Paired analysis confirmed a consistent reduction in L5-LDL across individual samples (*p* = 0.01). These results indicate that sialic acid residues contribute to the electronegative phenotype of VLDL and LDL particles. While the present data support a role for apolipoprotein sialylation in modulating lipoprotein charge properties, the relatively small sample size represents a limitation, and larger studies will be required to confirm the generalizability of these findings and to further define the contribution of specific apolipoproteins to L5-LDL formation.

### Elevated LPC levels are associated with hepatic ApoE glycosylation

**Figure 3:**
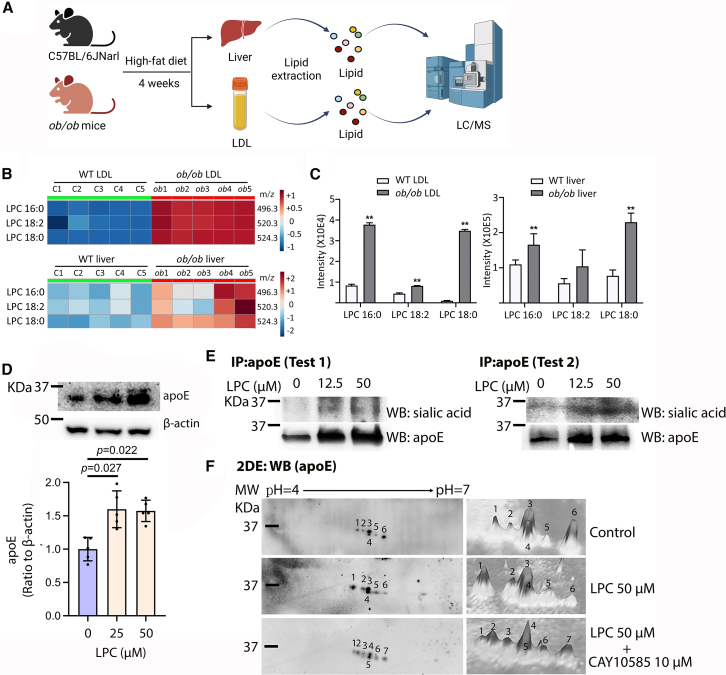
Elevated LPC in *ob/ob* mice is associated with increased hepatic ApoE glycosylation (A–C) Lipidomic analysis of liver and LDL from C57BL/6 wild-type mice (*n* = 5) and *ob/ob* mice (*n* = 5) fed a high-fat diet for 4 weeks. LC-MS^E^ identified LPC 16:0 (m/*z* 496.3), LPC 18:2 (m/*z* 520.3), and LPC 18:0 (m/*z* 524.3) as the major species, all of which were significantly increased in both liver tissue and circulating LDL from *ob/ob* mice compared with controls (*p* < 0.01). (D) Huh-7 hepatocytes treated with 25 or 50 μM LPC for 24 h exhibited a dose-dependent increase in apoE protein abundance (*n* = 5). (E) Immunoprecipitated apoE from LPC-treated hepatocytes showed enhanced sialylation detected by western blot analysis (*n* = 2). (F) Two-dimensional electrophoresis (isoelectric focusing followed by SDS-PAGE) of apoE (∼35 kDa) revealed a shift toward more acidic isoforms (lower pI) after 50 μM LPC exposure, consistent with increased *O*-glycosylation and sialylation. This acidic shift was attenuated by co-treatment with the HIF-1α inhibitor CAY10585. Data are expressed as mean ± SD. Statistical significance was assessed using the Mann-Whitney U test (∗*p* < 0.05; ∗∗*p* < 0.01).

Elevation of hepatic LPC has been associated with MetS,^18^ yet the mechanisms connecting LPC accumulation to lipoprotein dysfunction remain unclear. In our cohort, LPC levels within LDL were significantly higher in MetS patients than in healthy controls (Figure S1). To model hepatic LPC accumulation, we used *ob/ob* mice, a well-established MetS model.^33^^,^^34^ LC-MS^E^ analysis identified LPC 16:0 (m/*z* 496.3), LPC 18:0 (m/*z* 524.3), and LPC 18:2 (m/*z* 520.3) as the predominant LPC species, all of which were significantly elevated in both liver tissue and LDL fractions from *ob/ob* mice compared with controls (Figures 3A–3C; *n* = 5/group, *p* < 0.01).

In Huh-7 hepatocytes, LPC treatment reduced cell viability in a dose-dependent manner (Figure S2; *n* = 39 measurements pooled from independent experiments, *p* < 0.0001) but substantially increased apoE expression (Figure 3D; *n* = 5, *p* < 0.05). Immunoprecipitated apoE from LPC-treated cells showed enhanced sialylation (Figure 3E; *n* = 2). Two-dimensional gel electrophoresis revealed a leftward shift of apoE toward a more acidic isoelectric point, indicative of increased electronegativity; this shift was reversed by co-treatment with the HIF-1α inhibitor CAY10585 (Figure 3F; *n* = 1). Together, these findings suggest that LPC exposure promotes apoE glycosylation in hepatocytes.

### LPC induces HIF-1α-dependent upregulation of GALNT2 and ST3GAL1

**Figure 4:**
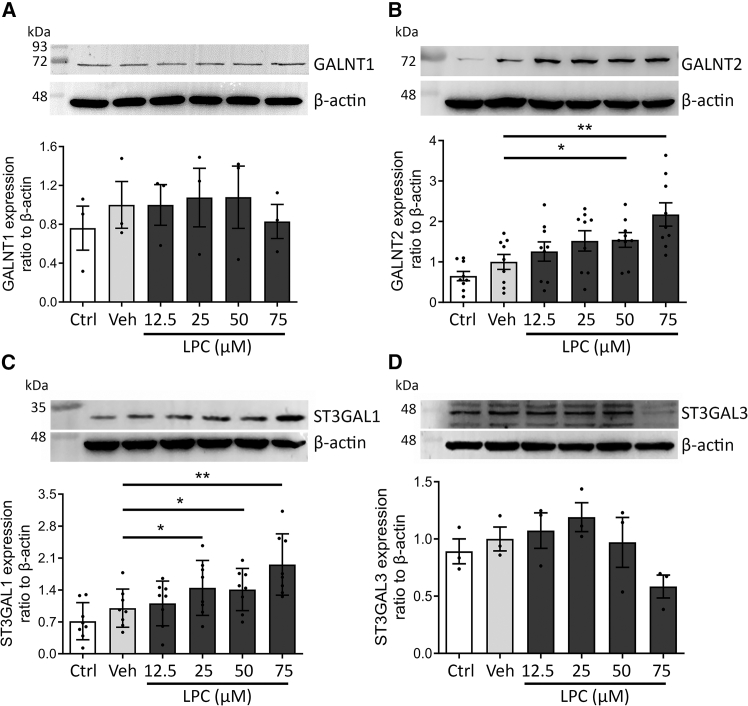
LPC selectively induces GALNT2 and ST3GAL1 expression in hepatocytes Huh-7 cells were treated with increasing concentrations of LPC for 24 h, and *O*-glycosylation-related enzymes were analyzed by western blotting (*n* = 3–9 independent experiments). Densitometric quantification was performed using ImageJ. (A) GALNT1 expression remained unchanged across LPC doses (*n* = 3). (B) LPC induced a dose-dependent increase in GALNT2 expression (*n* = 9; 50 μM, *p* < 0.05; 75 μM, *p* < 0.01). (C) ST3GAL1 expression was significantly upregulated by LPC (*n* = 8; 25 and 50 μM, *p* < 0.05; 75 μM, *p* < 0.01). (D) ST3GAL3 expression was unaffected by LPC treatment (*n* = 3). Data are presented as mean ± SD from independent biological experiments. Statistical analyses were performed using the Mann-Whitney U test or one-way ANOVA, as appropriate.

Because *O*-glycosylation involves the sequential addition of *N*-acetylgalactosamine and sialic acid, we next examined whether LPC modulates the expression of key glycosyltransferases involved in this process. Huh-7 cells were treated with increasing concentrations of LPC (0, 12.5, 25, 50, and 75 μM) for 24 h. LPC significantly upregulated GALNT2 and ST3GAL1 expression in a dose-dependent manner (Figures 4B and 4C; *n* = 8–9, *p* < 0.05–0.001), whereas GALNT1 and ST3GAL3 levels remained unchanged (Figures 4A and 4D; *n* = 3). The extent of experimental replication was adjusted according to the magnitude and reproducibility of the observed effects, with additional independent experiments performed for targets showing consistent dose-dependent responses.

**Figure 5:**
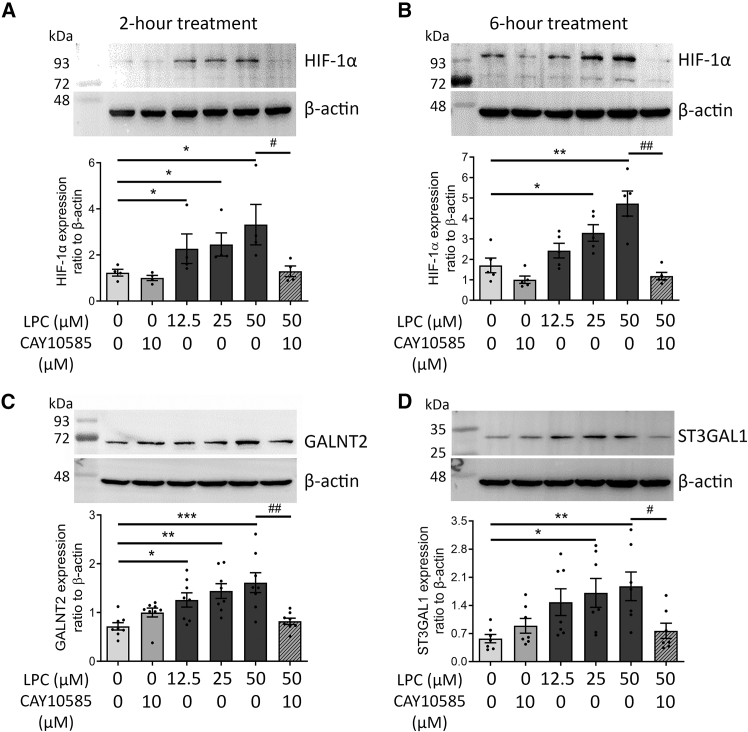
HIF-1α inhibition attenuates LPC-induced GALNT2 and ST3GAL1 expression in hepatocytes Huh-7 cells were treated with LPC for the indicated durations, with or without the HIF-1α inhibitor CAY10585. (A) LPC increased HIF-1α protein abundance after 2 h (*n* = 4, *p* < 0.05). (B) A further rise in HIF-1α levels was observed after 6 h (*n* = 5, *p* < 0.01), and this induction was completely blocked by CAY10585. (C) CAY10585 co-treatment significantly reduced LPC-induced GALNT2 expression (*n* = 8, *p* < 0.05–0.01). (D) LPC-stimulated ST3GAL1 upregulation was similarly suppressed by HIF-1α inhibition (*n* = 7, *p* < 0.05–0.01). Data are presented as mean ± SD. Statistical analyses were performed using the Mann-Whitney U test or one-way ANOVA, as appropriate.

LPC treatment also induced a rapid increase in HIF-1α protein at 2 and 6 h, an effect completely blocked by the HIF-1α inhibitor CAY10585 (Figures 5A and 5B; *n* = 4 for 2 h, *n* = 5 for 6 h, *p* < 0.05). Consistently, CAY10585 co-treatment suppressed LPC-induced GALNT2 and ST3GAL1 expression (Figures 5C and 5D; *n* = 7–8, *p* < 0.05). These findings suggest that LPC upregulates key glycosyltransferases through HIF-1α-dependent signaling, thereby promoting ApoE *O-*glycosylation.

### LPC enhances ApoE sialylation through HIF-1α signaling

**Figure 6:**
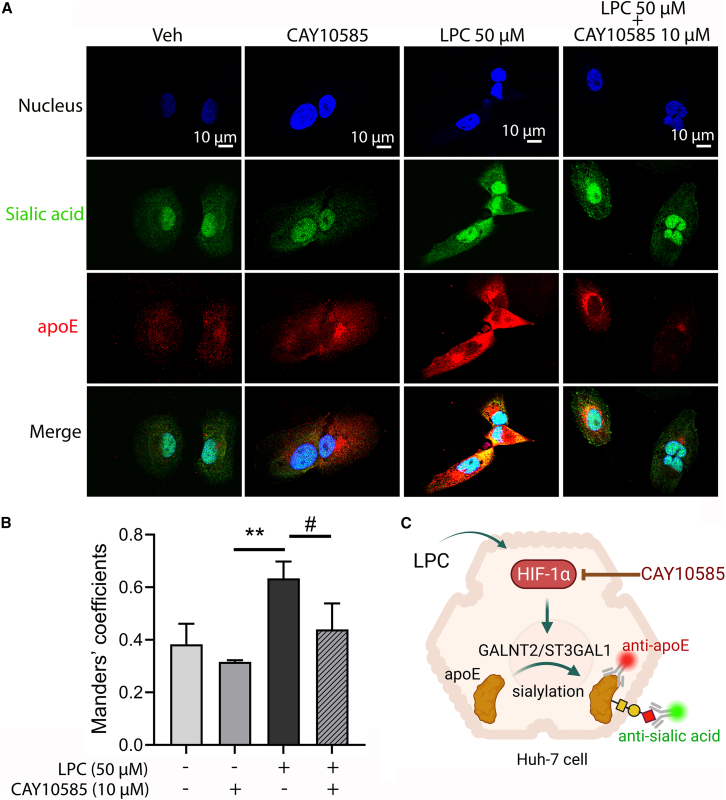
LPC promotes apoE sialylation in an HIF-1α-dependent manner in hepatocytes Huh-7 cells were treated with 50 μM LPC for 24 h with or without the HIF-1α inhibitor CAY10585 (10 μM). (A) Representative confocal images showing apoE (red), sialic acid (green), and nuclei (blue, DAPI). LPC treatment increased both apoE abundance and sialic acid signal. (B) Manders’ coefficient analysis demonstrated a significant rise in apoE-sialic acid colocalization following LPC exposure (0.38–0.63), which was partially reduced by HIF-1α inhibition (0.44; *n* = 3 per condition). (C) Schematic overview illustrating that LPC-induced apoE sialylation requires HIF-1α activity. Data are presented as mean ± SD from independent biological experiments. Statistical analyses were performed using the Mann-Whitney U test or one-way ANOVA. ∗∗*p* < 0.01 vs. CAY10585 alone; #*p* < 0.05 vs. LPC.

To determine whether the LPC-HIF-1α axis regulates apoE glycosylation, Huh-7 cells were treated with 50 μM LPC for 24 h with or without the HIF-1α inhibitor CAY10585 (10 μM). Confocal microscopy showed that LPC markedly increased sialic acid accumulation (green) and ApoE expression (red) in Huh-7 cells (Figure 6A).

Colocalization analysis using Manders’ coefficients with Costes’ automatic thresholding revealed limited overlap between apoE and sialic acid in control cells (coefficient = 0.38). LPC treatment significantly increased colocalization to 0.63, indicating enhanced apoE sialylation. This increase was partially reversed by CAY10585, which reduced the coefficient to 0.44, demonstrating that LPC-induced apoE sialylation requires HIF-1α signaling (Figure 6B; *n* = 3, *p* < 0.05). Together, these results show that activation of the LPC-HIF-1α axis is essential for enhancing apoE sialylation (Figure 6C).

### LPC-induced glycosylated apoE released from hepatocytes colocalizes with LOX-1 on endothelial cells

Enhanced sialylation of hepatocyte-derived apoE has been reported to increase its electronegativity and influence its interaction with endothelial receptors, including LOX-1.^8^^,^^9^ To investigate whether LPC-induced apoE modification alters its spatial association with LOX-1 on endothelial cells, Huh-7 cells were treated with 50 μM LPC for 24 h in the presence or absence of the HIF-1α inhibitor CAY10585. CCM was collected and incubated with or without α2-3 neuraminidase (160 U) at 37°C for 2 h before application to bovine aortic endothelial cells (BAECs).

**Figure 7:**
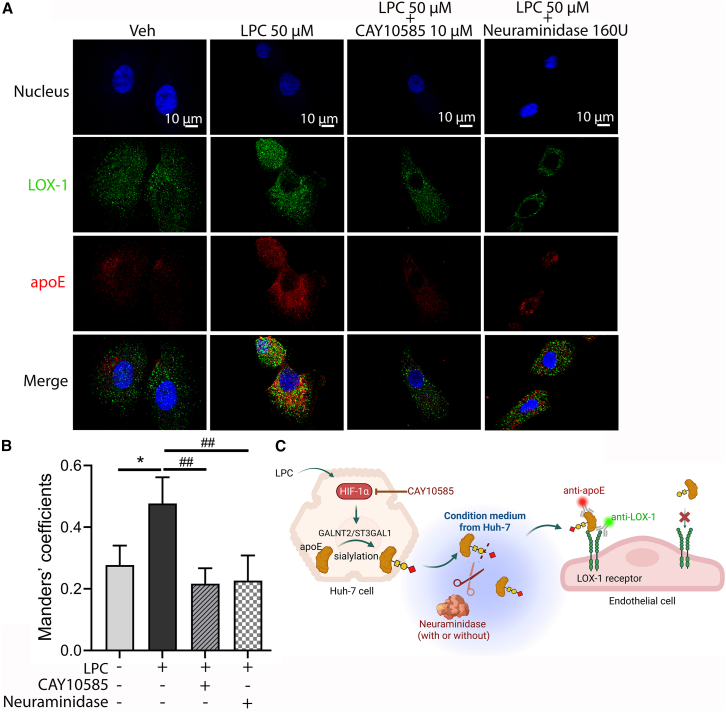
LPC-induced sialylated apoE released from hepatocytes shows increased colocalization with endothelial LOX-1 Huh-7 hepatocytes were treated with 50 μM LPC for 24 h with or without the HIF-1α inhibitor CAY10585 (10 μM). Conditioned culture medium (CCM) was collected, incubated with or without α2-3 neuraminidase (160 U, 2 h), and applied to BAECs for 30 min. (A) Representative confocal images showing apoE (red), LOX-1 (green), and nuclei (blue, DAPI). CCM from LPC-treated hepatocytes increased apoE colocalization with LOX-1 on the endothelial cell surface. (B) Quantification of apoE-LOX-1 colocalization using Manders’ coefficient. LPC treatment increased colocalization from 0.28 to 0.48, whereas HIF-1α inhibition or neuraminidase pretreatment reduced this association (0.22 and 0.23, respectively; *n* = 3–6 per condition). No apparent differences in overall LOX-1 fluorescence intensity were observed across groups under identical acquisition settings. (C) Schematic summary illustrating that LPC stimulates hepatocytes to release sialylated apoE, which exhibits enhanced association with endothelial LOX-1. Inhibition of HIF-1α signaling or removal of terminal sialic acids attenuates this interaction. Data are presented as mean ± SD from independent biological experiments. Statistical analyses were performed using the Mann-Whitney U test or one-way ANOVA. ∗*p* < 0.05 vs. control; ##*p* < 0.01 vs. LPC.

Confocal microscopy revealed that ApoE (red) present in CCM from LPC-treated hepatocytes exhibited prominent colocalization with LOX-1 (green) on the plasma membrane of BAECs within 30 min (Figure 7A). Manders’ coefficient analysis demonstrated increased ApoE-LOX-1 colocalization in BAECs exposed to CCM from LPC-treated cells (0.48) compared with control conditions (0.28). This colocalization was significantly reduced to 0.22 and 0.23 following CAY10585 co-treatment or α2-3 neuraminidase pretreatment, respectively (Figure 7B; *n* = 3, *p* < 0.05). Notably, no apparent differences in overall LOX-1 fluorescence intensity were observed across experimental conditions under identical imaging settings, supporting that the changes in colocalization primarily reflect altered apoE-LOX-1 interaction rather than differences in LOX-1 expression.

Because this experiment measures ligand-receptor spatial colocalization at the endothelial cell surface rather than receptor expression, these findings suggest that LPC-induced, HIF-1α-dependent apoE glycosylation promotes the association of hepatocyte-derived apoE with endothelial LOX-1 (Figure 7C).

## Discussion

MetS is characterized by insulin resistance, chronic inflammation, and oxidative stress, all of which accelerate cardiovascular disease progression.^35^ Electronegative LDL is increased in metabolic disorders and contributes to endothelial injury through impaired LDLR-mediated clearance and enhanced LOX-1 interaction, leading to endothelial apoptosis and vascular dysfunction.^36^^,^^37^^,^^38^ Consistent with these observations, we detected elevated V5-VLDL and L5-LDL, which have been shown to promote endothelial injury and adipocyte inflammation.^12^^,^^14^ Previous studies from our group further demonstrated that L5-LDL activates LOX-1-dependent signaling, resulting in inhibition of the PI3K/Akt pathway, mitochondrial dysfunction, and endothelial apoptosis.^9^ Despite strong evidence linking electronegative lipoproteins to atherogenesis, no current therapies specifically target their formation or removal.

Electronegative LDL is a multifactorial entity. Proposed mechanisms include oxidative modification, non-enzymatic glycation, enrichment of non-esterified fatty acids, alterations in lipid composition, incorporation of additional apolipoproteins such as apoCIII or apo(a), and conformational changes in apoB.^7^^,^^39^^,^^40^^,^^41^^,^^42^^,^^43^^,^^44^^,^^45^ Although lipoprotein(a) may contribute to LDL electronegativity due to its lower isoelectric point and partial overlap in density with LDL, our previous findings suggest that L5-LDL is not confined to Lp(a)-enriched fractions.^7^^,^^44^ Similarly, while oxidative modification has been implicated,^46^ prior studies indicate that lipid peroxidation alone does not fully account for the electronegative phenotype.^11^^,^^40^ Together, these observations support a model in which multiple structural and compositional changes contribute to LDL electronegativity.

Within this framework, our findings identify apoE sialylation as a contributing mechanism. ApoE functions as a ligand for LDLR-mediated clearance of apoB-containing lipoproteins, and increased sialylation may reduce its receptor affinity and promote lipoprotein persistence.^47^ We demonstrate that V5-VLDL, a hepatic precursor of L5-LDL, contains hyperglycosylated apoE at residues T194 and T289, suggesting that apoE modification occurs during VLDL assembly rather than exclusively in circulation. However, because apoB contains multiple glycosylation sites and neuraminidase affects several apolipoproteins, the relative contributions of apoE and other apolipoproteins (e.g., apoB or apoCIII) cannot be fully distinguished and warrant further investigation.

LOX-1 preferentially recognizes negatively charged lipoproteins through arginine-rich domains.^48^^,^^49^ Consistent with previous observations that sialylated apoE enhances electronegativity and LOX-1 binding,^7^^,^^8^ we found that apoE sialylation promotes apoE-LOX-1 interaction, which is attenuated by HIF-1α inhibition or neuraminidase treatment. Enrichment of hyperglycosylated apoE in V5-VLDL and L5-LDL further supports its contribution to the electronegative phenotype.

NAFLD (non-alcoholic fatty liver disease), the hepatic manifestation of MetS, is characterized by lipid remodeling and has been linked to LPC-enriched electronegative LDL, suggesting a liver-heart axis in vascular injury.^50^ Although the *ob/ob* mouse model does not fully recapitulate the human lipoprotein profile, it remains a useful model for studying hepatic metabolic dysregulation. In this study, *ob/ob* mice exhibited increased hepatic and circulating LPC levels, consistent with clinical lipidomic studies reporting elevated LPC species in MetS and their association with hypertriglyceridemia, obesity, and insulin resistance.^51^

To investigate hepatocyte-derived mechanisms, we used Huh-7 cells to examine LPC-induced modification of apolipoproteins. ApoE secreted from hepatocytes can exist in both lipid-poor and lipoprotein-associated forms, and LPC-induced modification may influence both its incorporation into lipoproteins and its interaction with cell surface receptors.^52^ However, the presence and quantitative levels of LPC-induced sialylated apoE in CCM were not directly measured. Thus, although apoE sialylation was observed in hepatocytes and functionally supported by endothelial cell-based assays, its extracellular distribution and mode of secretion remain to be clarified. In addition, the number of biological replicates varied across experiments, as replication was adjusted according to the magnitude and reproducibility of observed responses; increasing replication for non-responsive targets may further improve statistical robustness.

**Figure 8:**
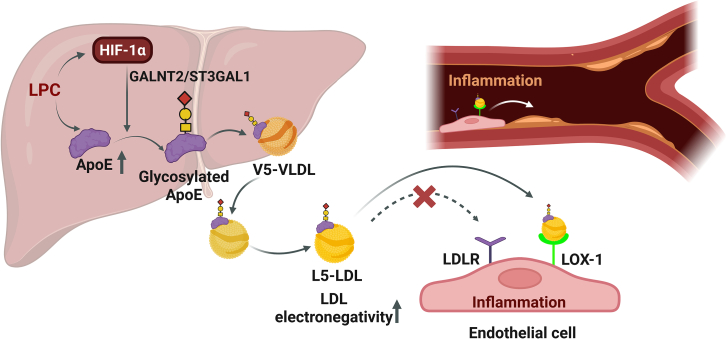
Working model illustrating the proposed mechanism linking hepatic LPC accumulation to apoE glycosylation and endothelial inflammation Based on the findings of the present study together with previously established mechanisms of electronegative lipoprotein biology, hepatic accumulation of LPC is proposed to activate HIF-1α signaling in hepatocytes. HIF-1α upregulates the glycosyltransferases GALNT2 and ST3GAL1, promoting *O*-glycosylation and sialylation of apoE. This modification results in the secretion of apoE-containing V5-VLDL particles with increased electronegativity. During lipolytic remodeling in the circulation, V5-VLDL can be converted to L5-LDL, which retains glycosylated apoE and exhibits enhanced surface electronegativity. Consistent with previous studies demonstrating receptor preference of electronegative lipoproteins, L5-LDL preferentially interacts with the scavenger receptor LOX-1 rather than LDLR on endothelial cells, thereby promoting inflammatory signaling and endothelial dysfunction. This working model provides a conceptual framework linking hepatic LPC dysregulation to the generation of electronegative lipoproteins and subsequent vascular injury in MetS.

Mechanistically, LPC activated HIF-1α and selectively upregulated GALNT2 and ST3GAL1, establishing an LPC-HIF-1α-glycosyltransferase axis. Inhibition of HIF-1α reduced apoE sialylation and attenuated apoE-LOX-1 interaction, supporting a functional link between hepatic lipid dysregulation and receptor-mediated vascular effects (Figure 8). Because glycosyltransferase activity may also influence apoB modification and lipoprotein secretion, this possibility warrants further investigation.

Together, our findings support a hepatic LPC-HIF-1α-apoE glycosylation axis that contributes to the formation of electronegative V5-VLDL and L5-LDL. This mechanism likely operates alongside previously described oxidative and compositional pathways, and apoE sialylation should be considered one component within a broader network regulating lipoprotein electronegativity in MetS.

In summary, this study identifies a regulated hepatic pathway that contributes to the formation of electronegative lipoproteins in MetS. We demonstrate that V5-VLDL, the precursor of L5-LDL, already contains hyperglycosylated apoE, indicating that this modification originates during hepatic lipoprotein assembly. Mechanistically, LPC activates HIF-1α and selectively induces GALNT2 and ST3GAL1, which promotes apoE sialylation and enhances LOX-1 interaction. These findings position hepatic LPC-driven apoE glycosylation within the broader multifactorial framework of LDL electronegativity. Although additional protein-specific studies are warranted, our data support a liver-centric regulatory pathway linking metabolic dysregulation to vascular injury. Targeting hepatic LPC metabolism, HIF-1α signaling, or apoE sialylation may therefore represent promising strategies to reduce electronegative lipoprotein burden and cardiovascular risk in MetS.

### Limitations of the study

This study has several limitations. First, the presence and quantitative levels of LPC-induced sialylated apoE in CCM were not directly measured, and its biochemical distribution between lipid-poor and lipoprotein-associated forms remains to be clarified. Second, although neuraminidase treatment supports a role for sialylation, it does not distinguish the relative contributions of apoE from other apolipoproteins such as apoB or apoCIII. Third, the number of biological replicates varied across experiments, reflecting differences in effect size and reproducibility, which may influence statistical robustness in certain assays. Finally, while the *ob/ob* mouse model recapitulates key aspects of metabolic dysregulation, it does not fully reproduce the human lipoprotein profile. In addition, although both male and female participants were included, the sample size was not sufficient to independently evaluate sex- or gender-specific effects. Future studies incorporating direct biochemical characterization and larger cohorts will be important to validate and extend these findings.

## Resource availability

### Lead contact

Further information and requests for resources and reagents should be directed to and will be fulfilled by the lead contact, Liang-Yin Ke (kly@kmu.edu.tw).

### Materials availability

This study did not generate new unique reagents.

### Data and code availability

• Data reported in this paper will be shared by the lead contact upon request.
• This paper does not report original code.
• Any additional information required to reanalyze the data reported in this paper is available from the lead contact upon request.

## Acknowledgments

We appreciate Prof. Shyi-Jang Shin, Chuan-Fa Chang, Chia-Yen Dai, Hsiang-Chun Lee, Wen-Lung Ma, Gopal Kedihithlu Marathe, Dr. Yen-Chun Chiu, and Shi Hui Law for their support.

This research was supported by grants from 10.13039/501100004694Kaohsiung Medical University, Taiwan (grant no. KMU-TC114A08 to L.-Y.K.); 10.13039/501100003835I-Shou University, Taiwan (ISU111-S-05 and ISU113-S-03 to H.-C.C.); 10.13039/100020595National Science and Technology Council, Taiwan (NSTC113-2314-B-214-002 to H.-C.C., NSTC109-2314-B-037-058 to C.-K.L., and NSTC113-2320-B-037-011-MY3 and NSTC114-2735-B-001-001 to L.-Y.K.).

## Author contributions

Conceptualization, Hua-Chen Chan and L.-Y.K.; methodology, Hua-Chen Chan, Hsiu-Chuan Chan, L.-F.W., Y.-M.K., and G.-M.K.; validation, M.-C.C., M.-L.Y., Hua-Chen Chan, and G.-M.K.; investigation and resources, M.-C.C., M.-L.C., W.-C.H., and C.-K.L.; formal analysis and data curation, Hua-Chen Chan and L.-Y.K.; writing – original draft, Hua-Chen Chan and L.-Y.K.; writing – review and editing, D.B., Hua-Chen Chan, and L.-Y.K.; visualization, Hua-Chen Chan and L.-F.W.; supervision, L.-Y.K.; project administration and funding acquisition, Hua-Chen Chan, C.-K.L., and L.-Y.K. All authors have met the criteria for authorship of this manuscript and have given final approval for the version to be published.

## Declaration of interests

The authors declare no competing interests.

## STAR★Methods

### Key resources table

**Table:** 

REAGENT or RESOURCE	SOURCE	IDENTIFIER
**Antibodies**		

ApoE antibody	Abcam	Cat# ab1907
ApoE antibody	Abcam	Cat# ab1906
GALNT1 antibody	Abcam	Cat# ab69959
GALNT2 antibody	Abcam	Cat# ab140637
ST3GAL1 antibody	Signalway Antibody	Cat# 22094
ST3GAL3 antibody	Abcam	Cat# ab58553
HIF-1α antibody	GeneTex	Cat# GTX127309
β-actin antibody	Sigma-Aldrich	Cat# A5441
LOX-1 antibody	Invitrogen	Cat# PA5-109203
Sialic acid antibody	Cloud-Clone	Cat# PAS099Ge01

**Biological samples**		

Plasma	Human	human subjects

**Chemicals, peptides, and recombinant proteins**		

Lysophosphatidylcholine (LPC)	Avanti Polar Lipids	Cat# 855675
CAY10585 (HIF-1α inhibitor)	Cayman Chemical	Cat# 10010585
α2-3 neuraminidase	New England Biolabs	Cat# P0743S
RapiGest SF	Waters Corporation	Cat# 186001861
Dithiothreitol (DTT)	Sigma-Aldrich	Cat# D9779
Trypsin Gold	Promega	Cat# V5280
Iodoacetamide (IAM)	Sigma-Aldrich	Cat# I1149

**Experimental models: cell lines**		

Huh-7	BCRC (Taiwan)	JCRB0403
Bovine Aortic Endothelial Cells (BAECs)	BCRC (Taiwan)	N/A

**Experimental models: organisms/strains**		

*ob/ob* mice (leptin-deficient mutant)	National Laboratory Animal Center (Taiwan)	RMRC12001
C57BL/6JNarl (wild-type)	National Laboratory Animal Center (Taiwan)	RMRC11005

**Software and algorithms**		

Progenesis QI	Waters Corporation	https://www.waters.com
ProteinLynx Global Server (PLGS) v2.5	Waters Corporation	https://www.waters.com
Fiji (ImageJ)	NIH	https://imagej.net/software/fiji/
SPSS v19.0	IBM	https://www.ibm.com
UNICORN	Cytiva	https://www.cytivalifesciences.com
Empower software	Waters Corporation	https://www.waters.com

### Experimental model and study participant details

#### Ethical approval and statement

This was a continuous study based on our previous work published in 2017 (*J Clin Endocrinol Metab* 102(12):4615–4625). The Institutional Review Board (IRB) of Kaohsiung Medical University Chung-Ho Memorial Hospital, Taiwan, approved this study. Written informed consent was obtained from all participants.

#### Human subjects

Eight healthy volunteers (5 males and 3 females) and eight patients with metabolic syndrome (MetS; 5 males and 3 females), aged 35–55 years, were enrolled. MetS was defined according to established criteria [33], including abdominal obesity (waist circumference ≥102 cm in men or ≥88 cm in women) in combination with at least two of the following: triglycerides ≥150 mg/dL, blood pressure ≥130/85 mmHg, fasting glucose ≥100 mg/dL, or HDL-cholesterol <40 mg/dL in men (<50 mg/dL in women). All procedures were performed under standardized conditions to minimize variability and sample degradation. Venous blood was collected into EDTA-coated tubes (VACUETTE, Greiner Bio-One). Plasma was separated by centrifugation at 3,000 rpm for 10 min at 4°C and subjected to a second centrifugation under the same conditions to remove residual cellular debris. Plasma samples were supplemented with a protease inhibitor cocktail (Roche) and used for lipoprotein isolation. The study was not powered to assess sex- or gender-specific effects; therefore, the influence of sex or gender on the reported outcomes could not be independently analyzed and is acknowledged as a limitation.

#### Animals

All animal procedures were approved by the Institutional Animal Care and Use Committee of Kaohsiung Medical University (IACUC No. 102149) and conducted in accordance with institutional guidelines. Homozygous *ob/ob* mice and age-matched C57BL/6JNarl controls of both sexes were obtained from the National Laboratory Animal Center, Taiwan. At eight weeks of age, mice were placed on a high-fat diet (60% kcal fat; D12492, Research Diets Inc.) for four weeks. At study completion, animals were euthanized and liver and plasma samples were collected for analysis.

#### Cell culture

Human hepatoma Huh-7 cells (JCRB0403, distributed by BCRC, Taiwan) and bovine aortic endothelial cells (BAECs) were obtained from BCRC (Taiwan). Cells were cultured in DMEM (Gibco) supplemented with 10% FBS and 1% penicillin–streptomycin at 37°C in 5% CO_2_. Independent cell line authentication by STR profiling was not performed in this study. Cells were routinely tested to confirm the absence of mycoplasma contamination.

### Method details

#### Lipoprotein isolation and subfractionation

Lipoproteins were isolated by sequential density-gradient ultracentrifugation using potassium bromide to adjust density. Ultracentrifugation was performed at 45,000 rpm, 4°C, for 24 h per step (Optima ultracentrifuge, Beckman Coulter). VLDL (0.96–1.006 g/mL) and LDL (1.019–1.063 g/mL) were isolated and dialyzed against buffer A (20 mM Tris-HCl, 5 mM EDTA, pH 8.0). Lipoprotein subfractionation was performed as described previously [13; 34]. Subfractionation was performed using an ÄKTA FPLC system with an anion-exchange UNO Q12 column (Bio-Rad). Lipoproteins were eluted using a NaCl gradient (0–100%) and analyzed using UNICORN software. Subfractions (V1–V5, L1–L5) were concentrated using PEG 20,000, dialyzed, filtered (0.22 μm), and stored at 4°C. All fractionation procedures were performed under identical buffer conditions to ensure reproducibility.

#### Enzymatic desialylation and electronegativity analysis

As previously reported, enhanced sialylation contributes to increased L5 LDL electronegativity [8]. LDL was incubated with or without α2-3 neuraminidase (26 U; P0743S, New England Biolabs) at 37°C for 2 h in GlycoBuffer 1 (pH 5.5). Reaction pH was verified prior to enzyme addition. Samples were analyzed using an ACQUITY UPLC system (Waters) with an anion-exchange column (Protein Pak Hi Res Q). Elution was monitored at 280 nm, and electronegativity was quantified based on retention profiles corresponding to L1–L5 subfractions. Data were processed using Empower software.

#### Cell treatment and conditioned medium

Huh-7 cells were treated with LPC (12.5–75 μM; Avanti Polar Lipids, AL, USA) for 24 h. LPC was prepared in 0.01% bovine serum albumin (BSA) as a carrier, and vehicle-treated cells were included as controls. In selected experiments, cells were co-treated with 10 μM CAY10585 (10010585; Cayman Chemical, MI, USA), a small-molecule inhibitor that promotes degradation of HIF-1α and suppresses HIF-1–dependent transcriptional activity. Cell viability following LPC treatment was assessed using an MTT assay. Briefly, Huh-7 cells were treated with vehicle or LPC at the indicated concentrations for 24 h. MTT reagent was added according to the manufacturer’s instructions, and absorbance was measured at 570 nm. Cell viability was quantified relative to the vehicle-treated control group. For CM experiments, 500 μL of conditioned culture medium (CCM) was collected from Huh-7 cells treated with 50 μM LPC. For enzymatic desialylation, CCM was supplemented with 10× GlycoBuffer 1 (P0743S; New England Biolabs, MA, USA) to achieve a final reaction pH of 5.5, which was verified and is optimal for α2-3 neuraminidase activity. Samples were incubated with or without 160 U of α2-3 neuraminidase (P0743S; New England Biolabs, Japan) at 37°C for 2 h according to the manufacturer’s instructions. Control samples underwent identical buffer treatment without enzyme. Following incubation, CCM was concentrated using Amicon Ultra centrifugal filters with a 50 kDa molecular weight cutoff (Sigma-Aldrich, MO, USA) to remove residual LPC and reaction components prior to application to BAECs. All treatments and processing steps were performed under identical conditions across experimental groups to ensure comparability.

#### Two-dimensional gel electrophoresis (2-DE)

For two-dimensional gel electrophoresis, 160 μL of 1× lysis buffer containing Protein Solubilizer 2, 3.5 mM Tris base (pH 7.4), 1× protease inhibitor, 23 mM DL-dithiothreitol (D9779-1G; Sigma-Aldrich, St. Louis, MO, USA), bromophenol blue, and ultrapure water was added to each sample. Samples were loaded into a ZOOM IPG Runner Cassette (Thermo Fisher Scientific, Cambridge, MA, USA) and incubated overnight to allow passive sample rehydration and entry into the immobilized pH gradient strip. Isoelectric focusing was then performed using an IPG Runner system (Thermo Fisher Scientific) under the following stepwise voltage conditions: 200 V for 20 min, 450 V for 20 min, 750 V for 20 min, 1200 V for 60 min, 1600 V for 60 min, and 1800 V for 60 min. After focusing, strips were sequentially equilibrated in equilibration buffer and alkylation buffer for 15 min each. The equilibrated strips were then subjected to second-dimension SDS–PAGE using Novex 4–20% Tris-Glycine ZOOM mini gels (1.0 mm; Thermo Fisher Scientific) in an XCell SureLock Mini-Cell system according to the manufacturer’s instructions.

#### Western blot analysis

Lipoprotein subfractions and cell lysates were prepared in RIPA buffer (VWR, PA, USA) supplemented with protease inhibitors (Sigma-Aldrich, MO, USA) on ice. Protein concentrations were determined using the BCA Protein Assay Kit (Thermo Fisher Scientific, MA, USA). Equal amounts of protein were denatured at 95°C for 5 min, separated by 10% SDS–PAGE at 70 V for 40 min followed by 100 V for 60 min, and transferred onto PVDF membranes (Merck Millipore, MA, USA). Membranes were blocked with 5% non-fat milk at 4°C overnight and incubated with primary antibodies diluted 1:1000 against ApoE (ab1906 and ab1907; Abcam), GALNT1 (ab69959; Abcam), GALNT2 (ab140637; Abcam), ST3GAL1 (#22094; Signalway Antibody), ST3GAL3 (ab58553; Abcam), HIF-1α (GTX127309; GeneTex), and β-actin (A5441; Sigma-Aldrich). After washing, membranes were incubated for 1 h at room temperature with HRP-conjugated secondary antibodies, including anti-rabbit IgG (1:5000; GTX213110-01; GeneTex) or anti-mouse IgG (1:5000; #20101; Leadgene, Taiwan). Signals were detected using an enhanced chemiluminescence substrate (JT96-K004M; T-Pro Biotechnology, Taiwan) and visualized using a Fusion Solo S imaging system (Vilber Lourmat, Germany). Band intensities were quantified using ImageJ and normalized to β-actin where appropriate.

#### Immunoprecipitation

Huh-7 cells treated with LPC were lysed in RIPA buffer supplemented with protease inhibitors (Sigma-Aldrich, MO, USA) on ice. Cell lysates were briefly sonicated to reduce viscosity and clarified by centrifugation at 12,000 × g for 10 min at 4°C. Protein concentrations were determined using a BCA Protein Assay Kit (Thermo Fisher Scientific, MA, USA). Equal amounts of total protein were incubated overnight at 4°C with anti-ApoE antibody (Abcam) under gentle rotation. Immune complexes were captured by incubation with protein A/G magnetic beads (Thermo Scientific Pierce) for 2 h at room temperature. Beads were then washed three times with wash buffer (25 mM Tris-HCl, 0.3 M NaCl, 0.05% Tween 20, pH 7.4) to remove non-specifically bound proteins. Bound proteins were eluted by heating the beads at 95°C for 5 min in SDS elution buffer (2% SDS, 50 mM Tris-HCl, pH 6.8, 10% glycerol, 5% β-mercaptoethanol, and 0.01% bromophenol blue). Eluted samples were subjected to subsequent Western blot analysis. All steps were performed under consistent conditions across experimental groups to ensure comparability.

#### Peptide digestion

Protein samples were dried under a nitrogen stream (Thermo Fisher Scientific, Cambridge, MA, USA) and resuspended in 100 μL RapiGest SF (186001861; Waters Corp., Milford, MA, USA) containing 5 mM dithiothreitol (DTT; Sigma-Aldrich, St. Louis, MO, USA). Samples were incubated at 60°C for 30 min to reduce disulfide bonds, followed by alkylation with 20 μL iodoacetamide (I1149; IAM; Sigma-Aldrich) in the dark for 30 min at room temperature. The reaction mixture was transferred to Amicon Ultra 0.5 mL centrifugal filters (50 kDa molecular weight cutoff; Merck Millipore, MA, USA) and centrifuged at 14,000 rpm for 5 min at 4°C. The retained protein fraction was washed six times with 450 μL ammonium bicarbonate (Sigma-Aldrich) under the same centrifugation conditions to remove low-molecular-weight contaminants and excess reagents. Proteins were then digested overnight at 37°C with trypsin gold (V5280; Promega, USA; final amount 3 μL of 0.1% solution). Following digestion, samples were acidified with 1 μL formic acid and incubated at 37°C for 45 min to terminate digestion and promote RapiGest degradation. The resulting peptide solution was passed through a 0.22 μm PVDF filter prior to liquid chromatography–mass spectrometry (LC-MS) analysis.

#### Liquid chromatography–mass spectrometry analysis of glycosylation

Glycosylation analysis was performed using a nano-flow UPLC system coupled to a Synapt High-Definition Mass Spectrometer (HDMS; Waters Corp., Milford, MA, USA) equipped with a nano-electrospray ionization (nano-ESI) source. Tryptic peptides were first loaded onto a C18 trap column and subsequently separated on a reversed-phase analytical column using buffer A (0.1% formic acid in water) and buffer B (acetonitrile containing 0.1% formic acid; LC/MS grade, Merck Millipore, MA, USA). Mass spectrometric data were acquired in data-independent acquisition mode (MSE), alternating between low collision energy (4 eV) and elevated collision energy (15–45 eV) to obtain both precursor ion and fragmentation information within a single analytical run. Spectra were collected over an m/z range of 50–2000. Glu^1^-fibrinopeptide B was used as a lock mass calibrant to maintain mass accuracy throughout the analysis. Raw data files were processed using ProteinLynx Global Server (PLGS) version 2.5 (Waters Corp., Milford, MA, USA). Glycopeptides were identified based on precursor mass, fragmentation patterns, and sequence assignment of b- and y-ions, together with mass shifts corresponding to glycan-associated modifications. Site-specific glycosylation of ApoE peptides was determined by manual interpretation of the annotated spectra in combination with PLGS-based peptide identification.

#### Lipid extraction

Liver tissue samples (30 mg) and LDL samples (30 μg total protein) were homogenized in 800 μL ice-cold water. For LDL, the protein amount was determined prior to extraction using a BCA Protein Assay Kit (Thermo Fisher Scientific, MA, USA). Methanol (2 mL; LC/MS grade, Thermo Fisher Scientific, MA, USA) and chloroform (1 mL; LC/MS grade, J.T. Baker, NY, USA) were added sequentially, followed by thorough mixing. An additional 1 mL chloroform and 1 mL water were then added to induce phase separation. Samples were centrifuged at 3,000 rpm for 10 min at 4°C. The lower organic phase was carefully collected, evaporated under a nitrogen stream, and reconstituted in a solvent mixture of isopropanol/acetonitrile/water (2:1:1, v/v/v; LC/MS grade, Merck Millipore, MA, USA; J.T. Baker, NY, USA) for subsequent LC-MS analysis. All extraction procedures were performed under identical conditions to minimize technical variation.

#### Liquid chromatography–mass spectrometry (LC-MS) for lipidomics

Lipid separation was performed using an ACQUITY UPLC system (Waters Corporation, Milford, MA, USA) equipped with a Charged Surface Hybrid (CSH) C18 column (Waters Corporation). The column temperature was maintained at 55°C and the flow rate was set at 0.1 mL/min. Mobile phase A consisted of acetonitrile/water (60:40, v/v) containing 10 mM ammonium formate and 0.1% formic acid, whereas mobile phase B consisted of isopropanol/acetonitrile (90:10, v/v) containing 10 mM ammonium formate and 0.1% formic acid. Lipid extracts were analyzed under these standardized chromatographic conditions prior to mass spectrometric detection. Mass spectrometric analysis was performed on a Xevo G2 QTof mass spectrometer (Waters Corporation) operating in positive electrospray ionization mode (ESI+). Instrument settings were as follows: capillary voltage, 2.0 kV; cone voltage, 30 V; source temperature, 120°C; desolvation temperature, 550°C; and collision energy, 35–55 V. Leucine enkephalin (m/z 556.2771) was used as a lock mass calibrant via the LockSpray interface to maintain mass accuracy throughout acquisition. Raw data were processed using Progenesis QI software (Waters Corporation). Lipid species were identified on the basis of retention time, accurate mass-to-charge ratio (m/z), and fragmentation patterns. LPC 16:0 (855675; Avanti Polar Lipids, AL, USA) was used as an internal standard for relative quantification of LPC species. All samples were analyzed under identical LC-MS conditions to minimize technical variation.

#### Immunofluorescence staining and colocalization analysis

Huh-7 cells or BAECs (1.6 × 10^4^ cells/well) were seeded onto glass coverslips in 24-well plates and cultured for 24 h before treatment. Following treatment, cells were washed with phosphate-buffered saline (PBS), fixed with 4% paraformaldehyde for 15 min at room temperature, and then permeabilized and blocked with 5% bovine serum albumin (BSA) containing 0.3% Triton X-100 for 1 h at room temperature. Cells were incubated overnight at 4°C with primary antibodies against sialic acid (1:500; PAS099Ge01, Cloud-Clone, Taiwan), ApoE (1:2000; ab1907, Abcam, UK), or LOX-1 (1:500; PA5-109203, Invitrogen, USA), diluted in 1% BSA/0.3% Triton X-100. After washing, cells were incubated for 1 h at room temperature with Alexa Fluor 488– or 546–conjugated secondary antibodies (1:500; Invitrogen, USA), counterstained with DAPI, and mounted using ProLong Gold Antifade Mountant. Images were acquired using a Zeiss LSM700 confocal microscope (White Plains, NY, USA). For colocalization analysis, 30 z-stacks were collected per sample at 1024 × 1024 pixel resolution with 0.2 μm intervals. Image acquisition parameters, including laser intensity, gain, and exposure settings, were kept constant across all experimental conditions. Manders’ coefficients were calculated using the JaCoP plugin in Fiji (ImageJ) with Costes’ automatic thresholding to quantify the fraction of ApoE signal overlapping with sialic acid or LOX-1. Analyses were performed across entire image fields without region of interest selection to avoid user bias. In Figure 7, the immunofluorescence analysis was used to assess ligand–receptor colocalization rather than to quantitatively determine LOX-1 expression levels.

### Quantification and statistical analysis

Data are presented as mean ± standard deviation (SD) from independent biological experiments unless otherwise specified. The number of biological replicates (n) for each experiment is indicated in the corresponding figure legends. The number of replicates was determined based on the consistency and magnitude of the observed responses across independent experiments. For experiments with small sample sizes (*n* ≤ 3), nonparametric statistical methods were applied to avoid assumptions of normal distribution. Differences between two groups were assessed using the Mann–Whitney U test or paired *t* test where appropriate. Comparisons among multiple groups were analyzed using one-way analysis of variance (ANOVA) or the Kruskal–Wallis test depending on data distribution, followed by appropriate post hoc tests. A *p*-value <0.05 was considered statistically significant. All statistical analyses were performed using SPSS software (version 19.0; IBM Corp., Armonk, NY, USA). All analyses were conducted using consistent parameters across experimental groups to ensure comparability.
