# Supplementary material for: The lysophosphatidylcholine-HIF-1α axis enhances apolipoprotein E sialylation and promotes electronegative LDL accumulation
Source: iScience. 2026 Jun 5;29(6):116167. doi: 10.1016/j.isci.2026.116167 (PMC13264260; doi:10.1016/j.isci.2026.116167)
Supplement: Document S1. Figures S1 and S2 [file mmc1.pdf]

iScience, Volume *29*

Supplemental information

The lysophosphatidylcholine-HIF-1α axis enhances apolipoprotein E sialylation and promotes electronegative LDL accumulation

Hua-Chen Chan, Hsiu-Chuan Chan, Liu-Fang Wang, Mei-Lin Chan, Wen-Chien Huang, Daniel Bender, Yu-Min Ko, Ming-Lung Yu, Guan-Ming Ke, Mei-Chuan Chou, Ching-Kuan Liu, and Liang-Yin Ke

Supplementary Information

Figure S1. LPC levels within LDL are significantly elevated in MetS patients (Related to Figure 2).

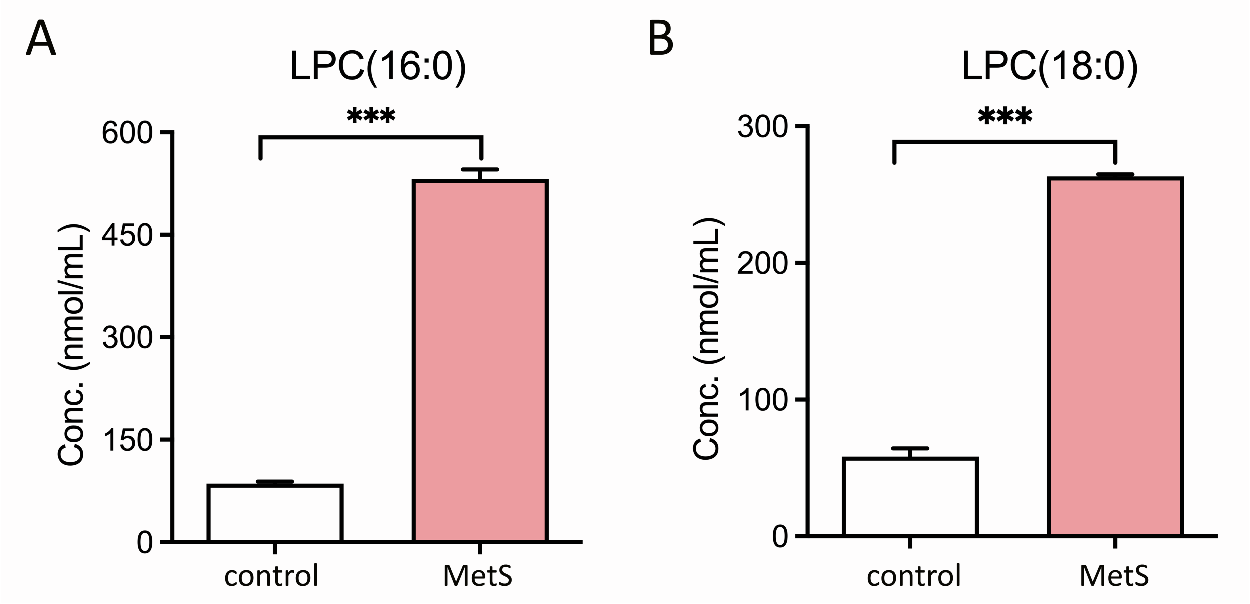

Figure S1. **LPC levels within LDL are significantly elevated in MetS patients (Related to Figure 2**). (A–B) Quantification of LPC species in LDL fractions isolated from healthy controls and patients with metabolic syndrome (MetS). LDL was isolated by ultracentrifugation. LPC(16:0) (A) and LPC(18:0) (B) were quantified by LC–MS using an Xevo G2 QTOF mass spectrometer (Waters Corporation). LPC standards (Avanti Polar Lipids) were used for calibration. Data are presented as mean ± SEM. Statistical significance was assessed using an unpaired two-tailed Student's t-test. ***$P < 0.001$.

Supplementary Information

Figure S2. LPC reduces cell viability in Huh-7 cells in a dose-dependent manner (Related to Figures 4 and 5)

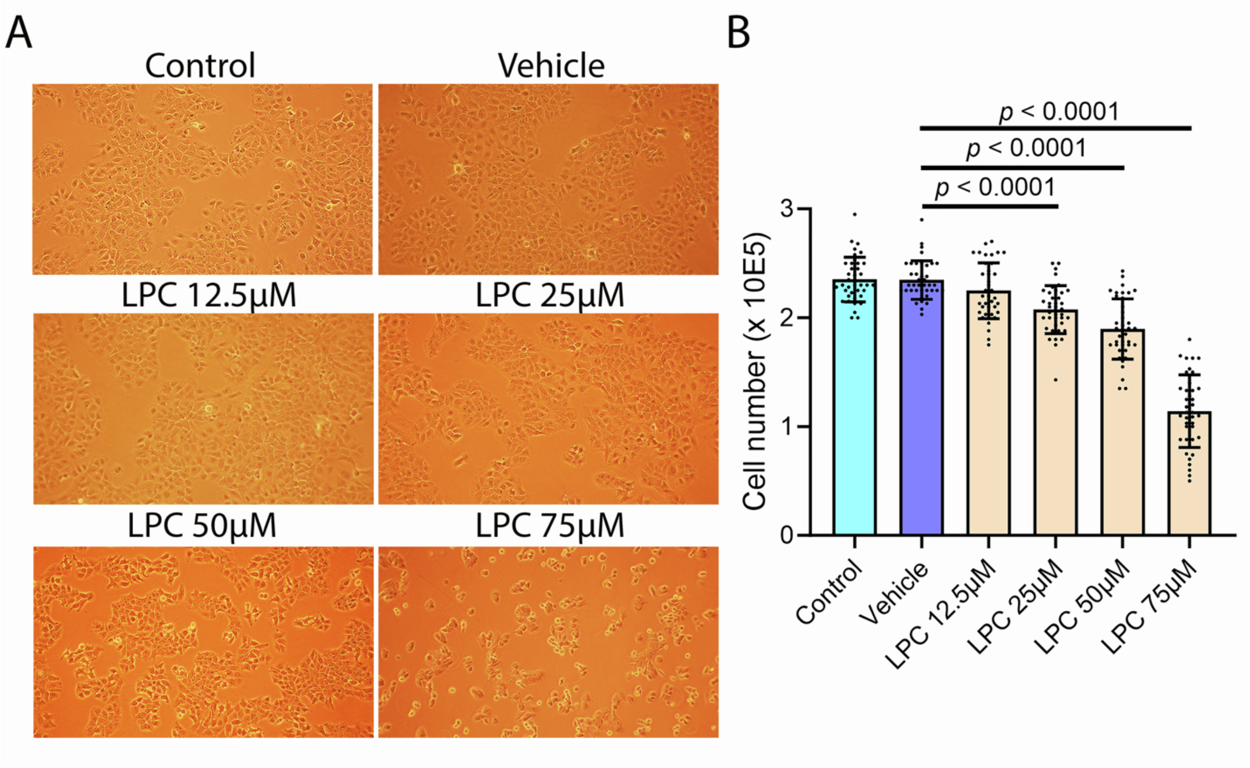

Figure S2. (A) Representative phase-contrast images of Huh-7 cells treated with vehicle or increasing concentrations of LPC (12.5–75 µM). (B) Cell viability was assessed by MTT assay following LPC treatment. Data are presented as mean ± SEM, with individual data points shown. Statistical significance was assessed using one-way ANOVA followed by a post hoc multiple comparisons test. Exact P values are indicated in the figure.
